# Development and Initial Assessment of a Patient Education Video about Pharmacogenetics

**DOI:** 10.3390/jpm7020004

**Published:** 2017-05-25

**Authors:** Rachel Mills, Megan Ensinger, Nancy Callanan, Susanne B. Haga

**Affiliations:** 1 Duke University Department of Medicine, Center for Applied Genomics and Precision Medicine, 304 Research Drive, Box 90141, Durham, NC 27710, USA; susanne.haga@duke.edu; 2 OhioHealth Genetic Counseling Program, Bing Cancer Center, 500 Thomas Ln Ste 2D Columbus, Columbus, OH 43214, USA; megan.ensinger@ohiohealth.com; 3 University of North Carolina, Greensboro School of Health and Human Services, Genetic Counseling Program, 1400 Spring Garden St. Greensboro, Greensboro, NC 27412, USA; npcallanan@uncg.edu

**Keywords:** patient education, educational video, pharmacogenetics

## Abstract

As few patient-friendly resources about pharmacogenetics are currently available, we aimed to create and assess a patient educational video on pharmacogenetic testing. A primary literature and resources review was conducted to inform the content and the format of the video. The educational video was then created using a commercially available animation program and pilot tested in focus groups of the general public and by an online survey of pharmacists. Emerging themes from the focus groups and survey indicate a desire for appropriate risk contextualization and specific examples when pharmacogenetic testing may be beneficial. Focus group participants also expressed a preference for a video with live action, and more text to reinforce concepts. Pharmacists generally felt that the video was understandable for patients and relevant for decision-making regarding testing. Using this initial feedback and the identification of important concepts to include in pharmacogenetics educational tools, we plan to revise the video, perform additional evaluations, and publish the video for public use in the future.

## 1. Introduction

Pharmacogenetic (PGt) testing analyzes genetic variants that affect drug targets, transport, and metabolism, contributing to the effectiveness of medication and risk of adverse drug reactions. The use of PGt testing has diffused across several clinical specialties and is a pillar of personalized and precision medicine [1]. There are many anticipated benefits to PGt testing including tailored treatment plans, reduced likelihood of adverse events, and improved outcomes. PGt testing also poses some limitations and risks including the potential for incidental results, inconclusive findings, and uncertain clinical utility. A clear and balanced presentation of these benefits and limitations is needed to enable patients to make an informed decision about undergoing PGt testing. However, limited pharmacist and physician knowledge about PGt testing [2,3], limited time during visits with providers, and limited patient resources create a challenging environment in which to introduce PGt testing.

Many of the currently available pharmacogenetic resources have been developed and published by PGt testing laboratories and may provide incomplete or biased information, or are specific to the laboratory’s unique testing services. Both primary care providers and pharmacists have expressed an interest in patient education materials to facilitate communication about testing [4,5].

Videos have been utilized for a number of years as an effective educational tool in healthcare, including for genetics services and testing. Use of videos has evolved from videotapes shown before or during clinical visits on televisions to web-based videos viewable from mobile devices such as smartphones and tablets. Educational videos may be used in healthcare classes for patients [6], within the public domain with advertisements that include an educational component [7], or in a clinical setting. Clinically, videos may address broad topics of interest to many patients (e.g., healthy diet) and be made available in an open setting, such as a waiting room or hall [8,9]. Alternatively, they may be integrated into patient care, providing detailed information about a particular condition [10] or serving as a decision aid regarding treatment or screening [11]. Educational videos have been as effective as an educational resource and/or decision aid in clinical genetics [12,13,14], and may be particularly useful in settings with few genetic counselors or providers who are unfamiliar with genetic testing [15]. For example, written materials and videos about genetic testing and results for cystic fibrosis carrier screening were equally effective in conveying information and were able to provide information without requiring face-to-face counseling [16]. The combined use of a video and counseling resulted in greater patient knowledge about positive newborn screening results for cystic fibrosis compared to counseling alone [17]. A decision aid video to guide patients’ decisions regarding testing for *p53* (cancer gene) lowered decisional conflict, increased knowledge, and decreased cancer worry [18]. Even for complex tests such as whole genome sequencing, patients found videos to be easy to understand and satisfactory while increasing their knowledge significantly [19]. Availability of educational resources outside of the clinical setting prior to interaction with the healthcare provider may facilitate discussion and the decision-making process [20,21]. Continued availability of these resources after a clinical visit can reinforce communication and improve patient understanding [22,23,24].

Given the many benefits of health educational videos and the lack of patient resources for PGt testing, a video about pharmacogenetics was developed to promote patients’ informed decision-making about testing. A first draft of the video was assessed by pharmacists and members of the general population through an online survey and focus groups, respectively. In this paper, we present our work on the development and the initial qualitative evaluation of an introductory video on PGt testing.

## 2. Results

### 2.1. General Public Focus Group Participants

Four participants attended the first focus group and three participants attended the second focus group for a total of seven participants. Due to the small groups, participant demographic information was not collected to protect participants’ identity, though both genders, multiple races, and a range of age groups were represented.

#### Focus group themes

Participant recommendations for video improvement were categorized into two broad categories: comments about content and comments about visual format. Within these broad categories, four major themes emerged: (1) specific examples of conditions/medications (content); (2) contextualizing risks of testing (content); (3) preference for live action (format); and (4) use of text to reinforce concepts (format).

Theme 1: Four participants suggested that specific and concrete examples of medications or conditions for which PGt testing would be applicable would be helpful to facilitate decisions about undergoing testing. Presenting specific applications for PGt testing could help them assign personal value to the test.

“*And like with the blood thinner and stuff like that, …I think including something like that into it would really, you know, help. So, maybe just include those [examples] so you can see the seriousness of what could happen if you take the wrong thing and you don’t have any testing. You know like the blood thinner, you could get a blood clot. You know, some kind of example like that would really push the seriousness of it.*”

“*It seems like [reasons for testing] may be pretty broad, but …if there are certain conditions, like … heart problems or digestive problems, …then this [testing] might be more targeted for you.*”

Theme 2: Four participants expressed concerns about segments of the video that discuss risks of testing such as genetic discrimination and felt that it would be more helpful to present statistics about cases of genetic discrimination, particularly in regard to PGt testing.

“*I do kind of like worry about everything all the time, so if I heard somebody like say, like this is something that somebody could steal your identity with, that would be pretty big cause for concern, but also like there are a lot of things that could happen, so I don’t know like if there is like a way that you can kind of add in an assurance, like this is extremely unlikely or like it’s probably not going to happen, something like that.*”

Those participants felt that the risks of PGt testing as portrayed in the video (with the cartoon character engaging in risky behavior—skydiving) were exaggerated when put in relative context of risks that they had experienced previously in their lives. In this frame, they did not perceive PGt testing as seriously risky.

“*That kind of led to believe there might be something seriously risky. I mean, out of all the things, somebody turning my information over is not the most riskiest thing I can think of in my life.*”

Theme 3: All seven participants expressed a desire for a video with real actors as opposed animation as they felt that the animation format minimized the seriousness of the subject matter. They felt that a serious topic like PGt testing ought to be portrayed with an equally serious presentation format.

“*This is a serious subject for me so I’d rather, in one sense I’d rather have real people doing it…I don’t have to be entertained.*”

“*For it to be kind of a little bit cheesy like that and kind of, you know, it takes away from I think the seriousness of the actual discussion.*”

Theme 4: Three participants indicated that including more text in the video would be helpful to reinforce or emphasize important concepts and would help maximize understanding.

“*I would’ve liked to see more text in the video. …especially like important key points or key phrases.*”

“*Yeah maybe, even like when somebody’s talking you can have the text under or something like that...just something to kind of drive the point home.*”

Other suggestions included incorporating information about panel testing for genes involved in drug response (as opposed to individual gene tests), creating a shorter video, giving patients print materials in addition to the video, using a more authoritative-sounding narrator, depicting individual differences in drug response with people holding various scales (e.g., high vs. low), reducing the volume of background music, and including information about family history of adverse drug response.

### 2.2. Pharmacist Survey Participants

Sixty-six members of the Community Pharmacists Pharmacogenetics Network (CPPN; www.rxpgx.com) were emailed an invitation to participate in the research survey and 10 respondents (15%) completed the survey in its entirety. Due to the small sample size, the demographic information collected was limited in order to protect participants’ identity. Reported characteristics of pharmacist respondents are summarized in Table 1. The pharmacist respondents worked in a range of practice settings; half of the respondents had not offered or applied PGt testing in the past twelve months (*n* = 5) and a majority had taken a course on pharmacogenetics (*n* = 7).

#### 2.2.1. Perspectives on Content and Patient Understandability 

When asked if the information in the video was comprehensive and complete, a majority (*n* = 7) of respondents strongly agreed or agreed. A majority of respondents (*n* = 7) also agreed that the information was relevant for a patient to make an informed decision about PGt testing. The majority of respondents (*n* = 7) felt that the information would likely be understandable for patients. Most respondents (*n* = 7) agreed that the tone of the video was appropriate for patients, and half of the respondents (*n* = 5) agreed that the video held their interest and attention. Statistical analysis to determine significance was not performed on this data due to the low number of responses.

Responses to the Patient Education Materials Assessment Tool (PEMAT) for audiovisual materials were scored individually and are summarized in Table 2. The educational video was given an average understandability score of 81% and an average actionability score of 78%.

#### 2.2.2. Recommendations for Improvement

Survey respondents were asked two open-ended response questions about what additional information, if any, should be added to the video and what other improvements could be made. Analysis of the open responses yielded two major themes: inclusion of specific examples of conditions/medications and contextualizing risks of testing.

Theme 1: Pharmacist respondents felt that it would be helpful to include examples of medications for which there is currently informative PGt testing to emphasize the utility of testing.

“*Seeing some real-life examples of medication situations helped by pharmacogenetics would be more impactful. The cartoon is nice and keeps things very basic so most patients will understand, but might not fully grasp the power of pharmacogenetics without a little more reality.*”

Theme 2: Some pharmacists felt that the risks of PGt testing were portrayed as overly negative and neglected to present the protections in place against genetic discrimination, even going so far as to indicate that information on the risk of genetic discrimination may be unnecessary to include as cases of genetic discrimination based on PGt testing are rarely observed.

“*The ‘risk’ should be reoriented to focus on the protections versus the risks. Stress the positive, don't scare patients off.*”

“*Remove the part about possibly having the test used against the patient...unless companies are doing this already. I have done over 1000 tests and not encountered this yet. As the language in the video sounds now I highly doubt many patients would choose to have the test done which negates so many benefits to a possible life-saving test.*”

Pharmacist respondents had a number of other suggestions to improve the video including adding information about the influence of drug transporters as well as drug metabolizing enzymes. Respondents also suggested including more information about benefits for patients undergoing PGt testing, selecting a different background music or lower volume relative to the narration, and creating an abbreviated version of the video to generate interest for a longer version.

## 3. Discussion

As PGt testing will likely be offered by providers with variable knowledge about pharmacogenetics, limited time with patients, and/or access to genetically-trained providers, additional educational approaches are needed to provide patients with the necessary information to make an informed decision and to understand how the test result may impact treatment decisions. With widespread access to digital media, short videos can be used to supplement patient-provider communication and enhance patients’ knowledge of this still relatively novel test. A comprehensive literature and resource review indicated a lack of patient educational materials about PGt, and few educational videos for commercial PGt tests. Though the available PGt videos provide some general background information, they are specific to a particular test offered by a commercial testing lab. Thus, we sought to create a universal educational video to introduce pharmacogenetics and PGt testing to patients. Feedback from a small group of the general public and pharmacists suggested that an educational video about PGt testing could be a useful tool to promote patient understanding and facilitate discussions about testing, though some revisions could help improve the initial version developed and shared with participants. 

Although animated videos have been effectively used to deliver health information [25], general public participants suggested that a live-action video may be more appropriate given the seriousness of the topic. Furthermore, with an actor, viewers may be able to better relate their own experiences or fears about adverse drug reactions. Several of the suggestions centered on how to improve patient understanding through use of text displayed on the video to highlight key points that may be lost or missed by the viewer. Graphics and other figures could be added to illustrate the multiple steps of how a drug moves through the body, the role of genes, and the testing process. Given the complexity of the scientific concepts and details about testing presented in the video, it is understandable that individuals from the general public desired additional features to be added to promote comprehension. While some similar challenges presenting complex scientific and medical concepts in an understandable language exist when developing any type of patient educational materials, videos must also consider aesthetics, the use of live actors, animation, and background scenes, and the use of graphics, figures, and text to enhance understanding.

Both general public participants and pharmacists made two similar suggestions. First, both groups suggested the addition of specific examples of PGt testing to enable patients to better understand how a test would be used for a patient in need of a certain medication. Inclusion of concrete examples of situations or conditions in which PGt testing would be used may help viewers relate to the personal benefits of testing. Pharmacists also suggested that inclusion of examples of medications for which there is informative PGt testing and information on the likelihood of new applications of PGt in the future may also be helpful to emphasize the utility of PGt testing. However, this finding is contradictory to another evaluation of an educational video about genetic testing and counseling which indicated that an educational video should provide broad information that may be applicable to a wider selection of patients and clinical indications [12].

The second suggestion made by both groups was to better balance the presentations of risks and benefits of testing. Some of the focus group participants felt the risk of testing was exaggerated and preferred for the risks to be presented relative to other types of risks of health interventions, such as risk of a serious adverse response to a medication or other physical harm. Pharmacists also suggested that information that would mitigate risk should also be presented, such as federal protections against genetic discrimination. As risk perception is very subjective, the appropriate framing of risk in a limited amount of time can be challenging. Overall, however, pharmacists felt that the information presented in the video was comprehensive, understandable, relevant for decision-making, and appropriate for patients.

### Study Limitations

Some limitations of the study should be acknowledged. The content of the video was developed by the synthesis of minimal existing literature on patient communication about PGt testing along with consensus by the research team. Therefore, the content of the educational video may not accurately reflect the informational needs of a given patient population. Our study is also limited by a small sample size and therefore our findings may not accurately represent the view of a larger population of patients or pharmacists. Furthermore, there may have been some ascertainment bias in the recruitment of participants in either the general public or pharmacist populations, particularly as several of the pharmacists were familiar with PGt testing.

## 4. Materials and Methods

We aimed to develop and conduct an initial qualitative assessment of an educational video about PGt testing to inform patients about the purpose, benefits, and limitations of testing. The initial version of the video was assessed by pharmacists and members of the general population through an online survey and focus groups, respectively. Information gathered from these initial assessments may be used to improve the video for future use in clinical and research settings.

### 4.1. Video Development

The purpose of the educational video is to provide general information for patients about pharmacogenetics, the process of PGt testing, risks and benefits of testing, and other relevant factors to consider before deciding whether or not to undergo testing. The script for the video was developed based on a literature review on pharmacogenetics, accessible online resources, and text included in a patient brochure that had been previously developed and assessed by patients for a clinical trial [26]. The content mirrors information that would be conveyed by a provider about a PGt test, though condensed for brevity. The developed script has a Flesch-Kincaid Reading Ease score of 55.7, which corresponds approximately to a ninth grade reading level. Inclusion of the term “pharmacogenetics,” which was defined at the beginning of the video, had a substantial effect on the readability of the script; when the term “pharmacogenetics” was removed, the Flesch-Kincaid Reading Ease score decreased to 62.7, dropping it to an eighth grade reading level. The final video is about five and a half minutes in length. It was created using the commercially available platform GoAnimate© (San Mateo, CA, USA), which is an easy-to-use program with a drag and drop interface for individuals without experience or extensive knowledge about graphic design or animation.

### 4.2. General Public Focus Group

The educational video was assessed via two focus groups of the general public; focus groups enable investigation of the perceptions and feelings of the general public regarding specific issues or services [27]. Focus groups participants were recruited from the general public in Durham, NC using Craigslist and flyers posted at Duke University Health System and within the Durham community. Additional participants were recruited from a population of patients who underwent PGt testing as part of a previous trial and agreed to be re-contacted for future research. Individuals had to be at least 18 years of age to participate.

A moderator guide was used to ensure consistency between the two group discussions; targeted questions were asked to direct feedback if necessary. The guide included questions and talking points focused on format (e.g., aesthetics, style, and length), understandability of content, and perceived utility. We showed the video in its entirety initially and replayed certain sections upon request to promote discussion, such as the sections on the risks of PGt testing, the effects of individual pharmacogenes on multiple drugs, and the importance of sharing PGt results with other prescribing providers.

### 4.3. Pharmacist Survey

The education video was also assessed using an online survey of pharmacists. Pharmacists who are members of CPPN were invited to view the video and provide feedback. A link to the video and a web-based survey was sent via email for ease of access as members of CPPN are located throughout the state of North Carolina. Reminder emails were sent one month after initial contact to increase response rate. The survey included measures assessing demographic and practice variables of the respondents. Questions regarding the content and format of the educational video included open-ended responses, and the PEMAT for audiovisual materials [28]. The PEMAT is a 15-question instrument used to evaluate and compare the understandability and actionability of patient education materials. Pharmacists also reported their level of agreement with five statements regarding the content and format of the educational video using a five-point Likert scale (Strongly Disagree–Strongly Agree) adapted from a six-question scale used in previous assessments of educational tools [29,30]. This scale was not validated in our study population prior to use. Pharmacists could also provide additional feedback about the video and suggestions for improvement in response to two open-ended questions (“What additional information should be added to the video?” and “Do you have any additional suggestions to improve this video?”).

### 4.4. Data Analysis

Focus group discussions were digitally recorded; recordings were transcribed and coded by themes by one author (M.E.). A second author (R.M.) coded transcripts independently and differences in coding were reviewed and discussed between the two until consensus was reached. Illustrative quotes were abstracted for each theme identified.

Summary statistics were calculated for each question. Responses to the PEMAT questions were scored according to the published guide to assess the understandability and actionability of the educational video [28]. One point was counted for each statement for which a respondent chose “Agree,” and 0 points were counted for each statement for which a respondent chose “Disagree,” giving a maximum possible score of 11 points for understandability and 4 points for actionability. Each respondent’s scores for understandability and actionability were converted to a percentage and all respondents scores were averaged to give an average understandability and actionability score for the educational video across all respondents.

The Institutional Review Boards of the Duke University Health System (Pro00067835) and the University of North Carolina at Greensboro (15-0452) approved the study.

## 5. Conclusions

Findings from the literature review as well as the focus groups and pharmacist surveys indicate that there is a need for PGt educational resources that focus on the risks and benefits of PGt testing. Therefore, providers offering PGt testing or supporting PGt services should consider the resources available and seek to provide additional information to supplement patient-provider discussions about testing. We envision the use of an introductory PGt video to supplement provider-patient discussion and potentially be accessible to patients outside of the clinic setting. A corresponding video reporting the results and the implications for treatment could also be developed to enhance comprehension of PGt test results relevant to the current as well as future treatments. Similar to what has been accomplished for at least one type of disease (inherited cancer [31]), a suite of PGt videos could be developed and made publicly accessible for a variety of content areas including general background, specific types of testing, and understanding the results. These short videos may reduce provider burden and promote patient awareness and informed decision-making by providing information about the purpose, risks, and benefits of PGt testing.

In summary, there is currently a lack of available resources for patients to learn about pharmacogenetics and PGt testing in general. We developed an educational video that pharmacist respondents believed to be understandable and that would guide patients in making an informed decision about testing. Both pharmacist and general public participants believed the video could be improved by including specific examples of drugs and PGt tests, and balancing the risks and benefits of testing. The feedback gathered will serve to inform the next iteration of the video in preparation for its implementation and evaluation in clinical settings.

## Figures and Tables

**Table 1:** Pharmacist respondent demographics.

	*n* = 10
**Primary Practice Environment**	
Community pharmacy	3
Academia	2
Long-term care	2
Industry	1
Other	2
**Degree**	
Bachelor‘s in pharmacy	2
PharmD	2
Other	4
Prefer not to answer	2
**Number of Years in Practice**	
0–9	3
10–19	0
20–29	2
30+	2
Prefer not to answer	3
**Use of PGt Testing in Preceding 12 Months**	
Have not used PGt testing	5
1–5 times	2
6–15 times	1
16+ times	2
**Completed A Course Focused on PGt**	
Yes	7
No	3

**Table 2:** Pharmacists’ The Patient Education Materials Assessment Tool for Audio-Visual Materials (PEMAT-A/V) Responses.

PEMAT-A/V Measurement Item	Disagree	Agree
The video makes its purpose completely evident.	2	8
The video uses common, everyday language.	2	8
Medical terms are used only to familiarize audience with the terms. When used, medical terms are defined.	1	9
The video uses active voice.	2	8
The video breaks or “chunks” information into short sections.	3	7
The video’s sections have informative headers.	3	7
The video presents information in a logical sequence.	1	9
The video provides a summary.	3	7
Text on the screen is easy to read.	1	9
The video allows the user to hear the words clearly (e.g., not too fast, not garbled).	1	9
The video uses illustrations and photographs that are clear and uncluttered.	2	8
The video clearly identifies at least one action the user can take.	1	9
The video addresses the user directly when describing actions.	2	8
The video breaks down any action into manageable, explicit steps.	2	8
The video explains how to use the charts, graphs, tables, or diagrams to take actions.	4	6

## Supplementary Materials

The first iteration of the video is viewable at https://youtu.be/AXi68Qp3hQ0.
